# Comparison of 4-Year Health Care Expenditures Associated With Roux-en-Y Gastric Bypass vs Sleeve Gastrectomy

**DOI:** 10.1001/jamanetworkopen.2021.22079

**Published:** 2021-09-09

**Authors:** Jean-Eric Tarride, Aristithes G. Doumouras, Dennis Hong, J. Michael Paterson, Semra Tibebu, Francis Nguyen, Richard Perez, Valerie H. Taylor, Feng Xie, Vanessa Boudreau, Eleanor Pullenayegum, David R. Urbach, Mehran Anvari

**Affiliations:** 1 Department of Health Research Methods, Evidence and Impact, Faculty of Health Sciences, McMaster University, Hamilton, Ontario, Canada; 2 Centre for Health Economics and Policy Analysis, McMaster University, Hamilton, Ontario, Canada; 3 Programs for Assessment of Technology in Health, The Research Institute of St. Joe’s Hamilton, St. Joseph’s Healthcare Hamilton, Hamilton, Ontario, Canada; 4 Division of General Surgery, McMaster University, Hamilton, Ontario, Canada; 5 Centre for Minimal Access Surgery, St Joseph’s Healthcare, McMaster University, Hamilton, Ontario, Canada; 6 ICES, Toronto, Ontario, Canada; 7 Institute of Health Policy, Management and Evaluation, University of Toronto, Toronto, Ontario, Canada; 8 Department of Family Medicine, McMaster University, Hamilton, Ontario, Canada; 9 Department of Psychiatry, University of Calgary, Calgary, Alberta, Canada; 10 The Hospital for Sick Children, Dalla Lana School of Public Health, University of Toronto, Toronto, Ontario, Canada; 11 Women’s College Hospital Research Institute, Department of Surgery, University of Toronto, Toronto, Ontario, Canada; 12 Women’s College Hospital Research Institute, Department of Health Policy, Management and Evaluation, University of Toronto, Toronto, Ontario, Canada

## Abstract

**Importance:**

Data on the long-term health care expenditures associated with bariatric surgery consisting of Roux-en-Y gastric bypass (RYGB) and sleeve gastrectomy are lacking.

**Objective:**

To compare 4-year health care expenditures after RYGB vs sleeve gastrectomy, identify factors independently associated with 4-year health care expenditures, and compare the procedures in terms of subsequent hospitalizations, bariatric procedures, and all-cause mortality.

**Design, Setting, and Participants:**

In this propensity score–matched cohort study, all residents of Ontario, Canada, who underwent publicly funded surgery with RYGB (n = 6301) or sleeve gastrectomy (n = 926) from March 1, 2010, to March 31, 2015, and consented to participate in the Ontario Bariatric Registry were eligible for the study. Follow-up was completed on March 31, 2019, and data were analyzed from May 5, 2020, to May 20, 2021.

**Interventions:**

RYGB and sleeve gastrectomy.

**Main Outcomes and Measures:**

Publicly funded health care expenditures, subsequent hospitalizations, bariatric procedures, and mortality during the 4 years after RYGB or sleeve gastrectomy.

**Results:**

The 1:1 matched study cohorts consisted of 1624 patients (812 per cohort) with a mean (SD) age of 48.0 (10.6) years, and 1242 women (76.5%). The mean body mass index (calculated as weight in kilograms divided by height in square meters) was 51.9 (8.3) for the RYGB cohort and 51.9 (8.9) for the sleeve gastrectomy cohort. The 4-year cumulative costs were not statistically significantly different between RYGB and sleeve gastrectomy (mean [SD], $33 682 [$31 169] vs $33 948 [$32 633], respectively; *P* = .86). Having a history of coronary artery disease was associated with a 35% increase in overall health care expenditures; chronic kidney disease, a 54% increase; and mental health admissions, a 67% increase. There were no statistically significant differences in all-cause mortality between RYGB and sleeve gastrectomy (1.5% vs 2.2%, respectively; *P* = .26) or the total number of hospitalizations (754 vs 669, respectively; *P* = .11) during the 4-year follow-up period. However, nonelective hospitalizations occurred more frequently with RYGB vs sleeve gastrectomy (472 vs 339, respectively; *P* = .002). Roux-en-Y gastric bypass was associated with relatively fewer subsequent bariatric procedures during the 4-year follow-up period (9 vs 40, respectively; *P* < .001).

**Conclusions and Relevance:**

In this Canadian population-based study, key results indicated that 4-year health care expenditures, all-cause mortality, and number of hospital admissions associated with RYGB did not significantly differ from those for sleeve gastrectomy. The rate of subsequent bariatric surgery was lower with RYGB. This study identified important patient-level drivers of health care expenditures that need to be further investigated.

## Key Points

**Question:**

Do the 4-year health care expenditures associated with Roux-en-Y gastric bypass (RYGB) differ from those associated with sleeve gastrectomy?

**Findings:**

In this population-based, matched cohort study of 1624 patients receiving either RYGB or sleeve gastrectomy in Ontario, Canada, there was no statistically significant difference in mean health care expenditures between RYGB and sleeve gastrectomy 4 years after the procedures ($33 682 vs $33 948, respectively).

**Meaning:**

These results may help to inform patients, surgeons, and policy makers on the relative values of RYGB and sleeve gastrectomy.

## Teaser

This propensity score–matched cohort study compares the 4-year health care expenditures of matched cohorts of patients undergoing Roux-en-Y gastric bypass vs sleeve gastrectomy and, secondarily, assesses factors independently associated with expenditures and compares them between procedures.

## Introduction

Recognition that pharmacotherapy and lifestyle changes alone will not produce clinically significant, sustainable weight loss has fueled increasing demand for bariatric surgery.^1^ Compared with no surgery, long-term randomized^2^ and nonrandomized^3^ evidence has shown durable outcomes over time after bariatric surgery. Five-year follow-up of 2 randomized clinical trials^4,5^ has also shown similar body weights, rates of type 2 diabetes remission, and reoperation rates among patients randomized to RYGB vs sleeve gastrectomy, the 2 most common bariatric procedures. These findings are in contrast to those from 2 large observational studies from the US^6,7^ that found relatively lower reoperation and reintervention rates with sleeve gastrectomy in the 5 years after bariatric surgery. However, few studies have compared the long-term health care expenditures associated with the 2 procedures. As such, the relative cost-effectiveness of the 2 procedures is unknown, which is an important gap in knowledge, because several countries (eg, Canada, United Kingdom, Australia) require both clinical and cost-effectiveness evidence for public reimbursement of health care technologies. The primary objective of this study was to compare the 4-year health care expenditures of matched cohorts of patients undergoing RYGB vs sleeve gastrectomy in a universal, publicly insured health care system. Our hypothesis was that health care expenditures would be similar in the 2 groups during the 4 years after surgery. Secondary objectives were to identify factors independently associated with 4-year health care expenditures and to compare RYGB and sleeve gastrectomy in terms of subsequent hospitalizations, bariatric procedures, and all-cause mortality.

## Methods

### Study Setting and Population

We undertook a population-based, matched cohort study of residents of Ontario, Canada, who underwent publicly funded bariatric surgery with RYGB or sleeve gastrectomy from March 1, 2010, to March 31, 2015, and who consented to participate in the Ontario Bariatric Registry. Briefly, the Ontario Bariatric Registry^8,9,10,11,12,13,14,15^ has collected real-world data since 2010 on all consenting patients eligible for publicly funded bariatric surgery in Ontario, Canada’s most populous province. In Ontario, individuals with a body mass index (BMI; calculated as weight in kilograms divided by height in meters squared) of at least 40 or a BMI of at least 35 with obesity-related comorbid conditions (eg, type 2 diabetes) are eligible for publicly funded RYGB, which is the primary bariatric surgery offered to patients with BMI of less than 60. Sleeve gastrectomy is also publicly reimbursed when RYGB is not possible owing to small-bowel disease and/or adhesions or previous surgery or when sleeve gastrectomy is performed as a planned staged surgery in patients with a BMI of greater than 60 to enable the patient to lose weight.^16^ As described elsewhere,^14,15^ patient-level records of the Ontario Bariatric Registry were linked with administrative health care records housed at ICES. These data sets were linked using unique encoded identifiers and analyzed at ICES, Toronto, Ontario. ICES is an independent, not-for-profit research institute whose legal status under Ontario’s health information privacy law allows it to collect and analyze health care and demographic data for health system evaluation, without individual patient consent. Owing to ICES status and because consent was required to participate in the Ontario Bariatric Registry, ethics approval was waived for this study by the Hamilton Integrated Research Ethics Board. The analyses and reporting follow the Strengthening the Reporting of Observational Studies in Epidemiology (STROBE) guidelines.^17^

ICES data include all health care services covered by the Ontario Ministry of Health and Long-Term Care, which is 100% of hospital care, emergency department visits, hospital-based outpatient specialty clinics, and physician visits for all residents of Ontario (40% of the Canadian population) and 100% of prescription drugs for residents 65 years and older and social assistance recipients. At the time of study, use of health care services and expenditure data were available through March 31, 2019, allowing a minimum of 48 months of follow-up for all patients undergoing bariatric surgery in our cohort.

### Primary and Secondary Outcomes

The primary study outcome was health care expenditures (detailed below) during the 4 years after bariatric surgery with RYGB or sleeve gastrectomy. Secondary outcomes were health care expenditures during 5 periods (index hospitalization, hospital discharge to the end of year 1, and years 2, 3, and 4 after the surgery), number of subsequent hospitalizations (ie, all types, elective, and nonelective), and bariatric procedures (ie, sleeve gastrectomy [Canadian Classification of Health Interventions procedure codes 1NF78GB or 1NF78WJ], RYGB [Canadian Classification of Health Interventions procedure codes 1NF78DQ or 1NF78SH], or duodenal switch [Canadian Classification of Health Interventions procedure codes 1NF78DI or 1NF78S]) as well as all-cause mortality.

### Statistical Analyses

Data were analyzed from from May 5, 2020, to May 20, 2021. As in previous work comparing health care expenditures associated with RYGB vs no surgery in Canada,^15^ we matched on a propensity score to create comparable cohorts of patients receiving RYGB vs sleeve gastrectomy. Variables included in the score were age, sex, BMI, year of surgery, geographical location (ie, 14 local health integration networks), census neighborhood income quintile, Ontario Marginalization Index,^18^ number of major Aggregated Diagnostic Groups (ADG; derived from the Johns Hopkins ACG System, version 10.0), potentially confounding chronic medical conditions derived from validated administrative data case definitions (eg, chronic kidney disease, coronary artery disease, type 2 diabetes, hypertension, hypercholesterolemia, and mood and anxiety disorders), total health care expenditures in the 5 years preceding the index surgery date, and number of days in the hospital and number of emergency department visits in the 365 days preceding the index date. We implemented greedy nearest-neighbor matching, which matches individuals based on the logit of their propensity score and surgical status (ie, RYGB or sleeve gastrectomy) using a caliper width of 0.2 of the SD. The cohorts were compared before and after 1:1 matching using standardized mean differences, where differences greater than 0.1 are generally considered meaningful.^19^ Patients were followed up from the date of their index bariatric surgery to a maximum of 4 years, censoring person-time at the point of loss of health insurance coverage or death. Health care expenditures expressed in 2018 Canadian dollars were calculated using standardized ICES costing algorithms^15,20^ for the total costs and for each cost component considered in the analyses.

To preserve the matched-pair nature of the data,^21^ we used generalized estimating equations (GEE) with a log link and an unstructured correction matrix to compare the 4-year cumulative health care expenditures associated with RYGB and sleeve gastrectomy as well as the health care expenditures incurred during the hospitalization associated with the index bariatric procedure, from discharge to end of year 1 and for each year thereafter. Multivariable GEE models controlling for age (<55 or ≥55 years), sex, surgery type (RYGB or sleeve gastrectomy), income quintiles, BMI categories (<50, 50-60, or >60), number of ADG diagnoses, and medical history before the index surgery (eg, chronic kidney disease, coronary artery disease, type 2 diabetes, hypertension, hypercholesterolemia, and mental health admissions) were used to identify factors associated with 4-year overall health care expenditures and 4-year costs associated with all-type hospitalizations, elective hospitalizations, nonelective hospitalizations, physician services (eg, primary care physicians, specialists, and laboratory tests), and other uses of health care services (eg, emergency department visits, same-day surgery, inpatient mental health, home care services, and hospital outpatient clinics). Generalized estimating equation models for count data (ie, using a negative binomial distribution) were used to compare the 2 procedures with respect to the number of subsequent hospitalizations and bariatric procedures during the 4-year period. McNemar tests for paired data were used for the 4-year comparison of all-cause mortality and the number of unique patients with at least 1 subsequent hospitalization or bariatric procedure. To help understand the generalizability of the results, matched and unmatched cohorts for each type of surgery were compared with respect to baseline characteristics. A 2-sided *P* < .05 was considered statistically significant. All analyses were performed at ICES using SAS Enterprise Guide, version 7.1 (SAS Institute, Inc; 2017). An intention-to-treat approach was used, in that patients remained in the group associated with their index bariatric procedure until such time as they lost health insurance coverage (eg, emigrated), died, or reached the end of the 4-year follow-up period. To comply with ICES privacy requirements, table cells for which fewer than 6 individuals contributed to the data were suppressed.

## Results

### Study Populations

After 1:1 propensity score matching, our study cohort consisted of 1624 matched patients (812 patients per group) receiving each surgery (mean [SD] age, 48.0 [10.6] years; 1242 women [76.5%] and 382 men [23.5%]). Mean (SD) age was 47.9 (10.6) years for the RYGB cohort and 48.1 years (10.6) years for the sleeve gastrectomy cohort (Table 1). Each cohort included 621 women (76.5%), 191 men (23.5%), and 310 patients with type 2 diabetes (38.2%). The mean (SD) BMI was 51.9 (8.3) for the RYGB cohort and 51.9 (8.9) for the sleeve gastrectomy cohort. eTable 1 in the Supplement presents the detailed characteristics of the patients undergoing RYGB (n = 6301) and sleeve gastrectomy (n = 926) before matching, whereas eTables 2 (RYGB cohort) and 3 (sleeve gastrectomy cohort) in the Supplement present the characteristics of the matched and unmatched cohorts for each type of surgery.

**Table 1.** Baseline Characteristics of the Matched Cohorts^a^

Characteristic	Treatment cohort^b^		Standardized mean difference
	RYGB (n = 812)	Sleeve gastrectomy (n = 812)	
Age, mean (SD), y	47.9 (10.6)	48.1 (10.6)	0.02
Age group, y			
<45	306 (37.7)	302 (37.2)	0.01
45-54	267 (32.9)	269 (33.1)	0.01
55-64	207 (25.5)	206 (25.4)	0.00
≥65	32 (3.9)	35 (4.3)	0.02
Sex			
Female	621 (76.5)	621 (76.5)	0.00
Male	191 (23.5)	191 (23.5)	0.00
BMI, mean (SD)	51.9 (8.3)	51.9 (8.9)	0.00
Year of index date			
2010	8 (1.0)	9 (1.1)	0.01
2011	96 (11.8)	88 (10.8)	0.03
2012	132 (16.3)	148 (18.2)	0.05
2013	285 (35.1)	276 (34.0)	0.02
2014	250 (30.8)	250 (30.8)	0.00
2015	41 (5.0)	41 (5.0)	0.00
Neighborhood income quintile			
First (lowest)	167 (20.6)	194 (23.9)	0.08
Second	211 (26.0)	191 (23.5)	0.06
Third	173 (21.3)	169 (20.8)	0.01
Fourth	146 (18.0)	149 (18.3)	0.01
Fifth (highest)	115 (14.2)	109 (13.4)	0.02
Marginalization index [summary score], mean (SD)^c^	3.04 (0.74)	3.07 (0.74)	0.03
Total No. of major ADGs, mean (SD)	6.81 (2.88)	6.80 (2.87)	0.00
Select medical conditions in the preceding 5 y			
CKD/ESKD	26 (3.2)	30 (3.7)	0.03
CAD/PCI/CABS	156 (19.2)	166 (20.4)	0.03
Type 2 diabetes	310 (38.2)	310 (38.2)	0.00
Hypertension	412 (50.7)	413 (50.9)	0.00
Hypercholesterolemia	135 (16.6)	133 (16.4)	0.01
Mood and anxiety disorders hospitalizations	7 (0.9)	15 (1.8)	0.09
Total health care expenditure in the preceding 5 y, mean (SD), CAD$	21 220 (7841)	23 428 (28 401)	0.08
Use of health care services in the preceding 365 d, mean (SD)			
No. of days in the hospital	0.48 (2.17)	0.50 (2.62)	0.01
No. of ED visits	0.91 (1.99)	0.91 (1.86)	0.00

Abbreviations: ADG, Johns Hopkins Aggregated Diagnostic Groups; BMI, body mass index (calculated as weight in kilograms divided by height in meters squared); CABS, coronary artery bypass surgery; CAD, coronary artery disease; CAD$, Canadian dollars; CKD, chronic kidney disease; ED, emergency department; ESKD, end-stage kidney disease; PCI, percutaneous coronary intervention; RYGB, Roux-en-Y gastric bypass.

^a^ In addition, the cohorts were matched according to the 14 local health integration networks of residence (data not shown).

^b^ Unless otherwise indicated, data are expressed as number (percentage) of patients.

^c^ Higher factor score represents a higher degree of marginalization.

In terms of follow-up, 97.4% of our study cohort achieved the minimum 48 months of follow-up (791 of 812 in each group). Of those lost to follow-up, 3 patients in the sleeve gastrectomy group and 9 patients in the RYGB group lost their health insurance status, whereas 18 patients in the sleeve gastrectomy group and 12 patients in the RYGB group died during the 4-year period. The 4-year all-cause mortality rate was 2.2% (18 of 812) for the sleeve gastrectomy group and 1.5% (12 of 812) for the RYGB group (*P* = .26).

### Health Care Expenditures

The mean (SD) health care expenditures during the 4 years after RYGB surgery were not significantly different from those after sleeve gastrectomy ($33 682 [$31 169] vs $33 948 [$32 633], respectively; *P* = .86). When the analyses were stratified by time, mean (SD) health care expenditures associated with the surgical admission were relatively higher for RYGB ($12 888 [$6404]) compared with sleeve gastrectomy ($12 231 [$5107]; *P* = .02). However, there were no statistically significant differences in mean (SD) health care expenditures between RYGB and sleeve gastrectomy from discharge to the end of year 1 ($5414 [$10 903] vs $5362 [$12 229]; *P* = .94), in year 2 ($5319 [$10 084] vs $5870 [$12 487]; *P* = .26), in year 3 ($5245 [$10 277] vs $5019 [$9549]; *P* = .66), or in year 4 ($4926 [$10 773] vs $5655 [$13 634]; *P* = .27) after the procedure. The Figure depicts these data, whereas Table 2 (RYGB) and Table 3 (sleeve gastrectomy) present the detailed cost data by year and cost components. Expenditures associated with hospitalization (RYGB, 47%; sleeve gastrectomy, 49%) and specialist visits (RYGB, 27%; sleeve gastrectomy, 24%) accounted for almost 75% of the 4-year health care expenditures associated with each procedure.

**Figure.**
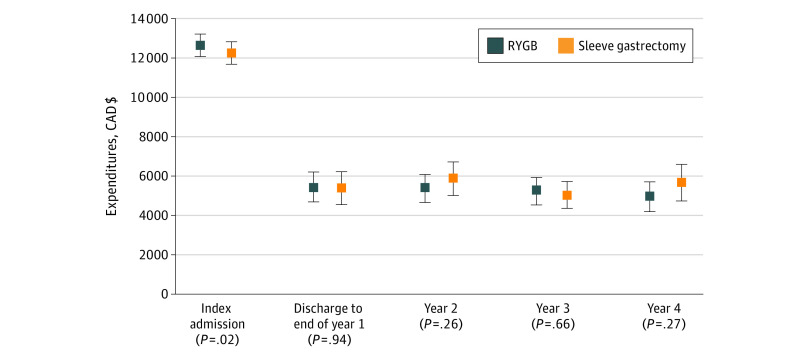
Mean (SD) Total Healthcare Expenditures of Roux-en-Y Gastric Bypass (RYGB) and Sleeve Gastrectomy by Study Periods Data are presented as mean expenditures (represented by dots) and 95% CIs (represented by error bars). CAD$ indicates Canadian dollars.

**Table 2.** Health Care Expenditures for Patients Undergoing RYGB by Year and Cost Components

Expenditure type	Mean (SD), CAD$					
	Index surgical admission (n = 812)	Discharge to end of year 1 (n = 812)	Time after index admission, y			4-y cumulative (n = 812)
			2 (n = 810)	3 (n = 806)	4 (n = 800)	
Total expenditures	12 888 (6404)	5414 (10 903)	5319 (10 084)	5245 (10 277)	4926 (10 773)	33 682 (31 169)
Inpatient	9105 (5866)	1659 (7349)	1733 (5562)	1609 (5605)	1659 (6953)	15 726 (17 909)
Elective hospitalizations	NA	322 (1733)	769 (3269)	781 (2639)	678 (2680)	11 622 (8337)
Nonelective hospitalizations	NA	1337 (6989)	965 (4175)	829 (4951)	982 (6300)	4104 (14 788)
Hospital outpatient clinics	49 (126)	704 (1299)	699 (1251)	681 (1275)	591 (1072)	2710 (4003)
Same day surgery	NA	275 (757)	363 (833)	339 (836)	295 (817)	1264 (1850)
ED	1 (25)	454 (811)	328 (608)	298 (628)	292 (635)	1366 (1985)
Home care services	19 (113)	272 (1348)	244 (1063)	267 (1125)	290 (1609)	1085 (3715)
Inpatient mental health	NA	95 (1851)	65 (1701)	215 (2690)	104 (1660)	486 (6234)
Laboratory	NA	377 (326)	211 (214)	177 (191)	143 (172)	905 (676)
Primary care physicians	16 (85)	259 (418)	246 (376)	266 (618)	272 (782)	1053 (1818)
Specialists	3697 (889)	1301 (2125)	1411 (2396)	1374 (2461)	1262 (2261)	9013 (7269)
Nonphysicians	NA	20 (45)	19 (57)	19 (80)	17 (36)	74 (145)

Abbreviations: CAD$, Canadian dollars; ED, emergency department; NA, not applicable; RYGB, Roux-en-Y gastric bypass.

**Table 3.** Health Care Expenditures for Patients Undergoing Sleeve Gastrectomy by Year and Cost Components

Expenditure type	Mean (SD), CAD$					
	Index surgical admission (n = 812)	Discharge to end of year 1 (n = 812)	Time after index admission, y			4-y cumulative (n =812)
			2 (n = 809)	3 (n = 803)	4 (n = 796)	
Total expenditures	12 231 (5107)	5362 (12 229)	5870 (12 487)	5019 (9549)	5655 (13 634)	33 948 (32 633)
Inpatient	9326 (4655)	1646 (9121)	2020 (8353)	1478 (5524)	2089 (10 193)	16 495 (20 445)
Elective hospitalizations	NA	369 (1998)	957 (3302)	930 (3833)	967 (3746)	12 468 (8807)
Nonelective hospitalizations	NA	1278 (8792)	1063 (7613)	548 (3716)	1123 (8981)	4026 (17 266)
Hospital outpatient clinics	63 (162)	758 (1311)	733 (1303)	657 (1133)	699 (1200)	2857 (3883)
Same day surgery	NA	263 (701)	353 (1067)	346 (858)	377 (918)	1327 (2055)
ED	3 (52)	352 (708)	266 (522)	267 (521)	275 (550)	1154 (1708)
Home care services	15 (102)	355 (1897)	426 (2091)	332 (1884)	315 (1437)	1432 (6026)
Inpatient mental health	NA	87 (1310)	169 (2415)	129 (1620)	63 (861)	445 (3778)
Laboratory	NA	407 (361)	205 (243)	180 (280)	144 (187)	929 (822)
Primary care physicians	12 (73)	275 (460)	250 (487)	234 (412)	262 (504)	1023 (1494)
Specialists	2812 (745)	1200 (1971)	1430 (2113)	1378 (3145)	1444 (2425)	8216 (7058)
Nonphysicians	NA	20 (68)	18 (39)	17 (34)	17 (32)	72 (118)

Abbreviations: CAD$, Canadian dollars; ED, emergency department; NA, not applicable.

During the 4-year follow-up, approximately 50% of patients had at least 1 readmission (RYGB, 370 of 812 [45.6%]; sleeve gastrectomy, 385 of 812 [47.4%]; *P* = .44) for a total of 754 and 669 hospital readmissions for the RYGB and sleeve gastrectomy cohorts, respectively (*P* = .11). Nonelective hospitalizations 4 years after the procedure occurred more often with RYGB vs sleeve gastrectomy (472 vs 339; *P* = .002), whereas there was no statistical difference in the number of subsequent elective hospitalizations between the procedures (282 vs 330, respectively; *P* = .07). More individuals underwent a second bariatric procedure when the index procedure was sleeve gastrectomy (37 of 812 [4.6%]) compared with RYGB (8 of 812 [1.0%]; *P* < .001) for a total of 40 and 9 subsequent bariatric procedures, respectively (*P* < .001). Among the sleeve gastrectomy group, 11 of 37 individuals who had a subsequent bariatric procedure had a BMI of greater than 60.

### Determinants of Health Care Expenditures

The results of the multivariable GEE regressions presented in Table 4 confirmed that there was no association between the type of bariatric surgery procedure and 4-year overall health care expenditures. However, sleeve gastrectomy was associated with a 7% increase in 4-year costs for elective hospitalization (rate ratio, 1.07; 95% CI, 1.01-1.14) and a 7% decrease in physician costs (rate ratio, 0.93; 95% CI, 0.87-0.99) after controlling for baseline characteristics. The GEE model of the costs associated with nonelective hospitalizations did not converge. Other results indicated that having a history of coronary artery disease (35% increase), chronic kidney disease (54% increase), and mental health admissions (67% increase) were the main factors contributing to the overall and individual cost components. Depending on the models, several other baseline characteristics were also associated with greater costs (eg, BMI of 50-59 and number of ADGs), but the magnitude of the increase was smaller. Table 4 provides the details of the regression analyses.

**Table 4.** Factors Associated With 4-Year Health Care Expenditures and per Major Cost Categories

Factor	Cost type, rate ratio (95% CI)^**a**^				
	Overall	Hospitalization	Elective hospitalization	Physician	Other
Sleeve gastrectomy (vs RYGB)	1.01 (0.94-1.09)	1.05 (0.96-1.16)	1.07 (1.01-1.14)^b^	0.93 (0.87-0.99)^b^	1.09 (0.97-1.22)
Male (vs female)	0.91 (0.83-1.00)	0.96 (0.85-1.07)	0.94 (0.87-1.02)	0.87 (0.81-0.93)	0.88 (0.75-1.03)
Age ≥55 y (vs <55 y)	1.08 (1.00-1.18)^b^	1.05 (0.94-1.17)	1.05 (0.97-1.12)	1.06 (0.99-1.13)	1.17 (1.02-1.33)^b^
BMI (vs <50)					
≥60	1.12 (0.99-1.25)	1.18 (1.03, 1.36)^b^	1.14 (1.04-1.26)^b^	1.06 (0.97-1.17)	1.06 (0.88-1.26)
50-59	1.09 (1.00-1.18)^b^	1.16 (1.04-1.28)^b^	1.10 (1.02-1.19)^b^	1.02 (0.95-1.09)	1.07 (0.94-1.22)
Income (vs lowest)					
Fifth quintile (highest)	0.98 (0.85: 1.12)	0.94 (0.80-1.11)	1.05 (0.94-1.16)	1.04 (0.91-1.18)	0.97 (0.80-1.18)
Fourth quintile	1.00 (0.87-1.14)	1.00 (0.83-1.19)	1.00 (0.89-1.13)	0.97 (0.88-1.08)	1.01 (0.84-1.22)
Third quintile	0.98 (0.87-1.09)	0.98 (0.86-1.13)	1.03 (0.94-1.12)	0.97 (0.88-1.0)	0.97 (0.81-1.16)
Second quintile	0.98 (0.88 1.09)	0.96 (0.84-1.11)	1.00 (0.91-1.09)	.98 (0.88-1.06)	1.03 (0.87-1.23)
Total No. of major ADGs	1.06 (1.04-1.08)^b^	1.04 (1.02-1.06)^b^	1.02 (1.01-1.03)^b^	1.06 (1.04-1.07)^b^	1.12 (1.09-1.15)^b^
5-y history					
CAD/PCI/CABS	1.35 (1.20-1.53)^b^	1.33 (1.14-1.56)^b^	1.05 (0.96-1.15)	1.30 (1.15-1.48)^b^	1.48 (1.25-1.75)^b^
Type 2 diabetes	1.07 (0.98-1.15)	1.07 (0.98-1.18)	0.99 (0.93-1.06)	1.10 (1.03-1.17)^b^	1.02 (0.89-1.15)
CKD/ESKD	1.54 (1.18-2.02)^b^	1.65 (1.18-2.31)^b^	1.35 (1.05-1.74)^b^	1.53 (1.24-1.88)^b^	1.32 (0.97-1.79)
Hyperlipidemia	1.05 (0.95-1.17)	1.05 (0.91-1.20)	1.01 (0.93-1.10)	1.10 (1.02-1.19)^b^	1.01 (0.86-1.18)
Mental health admissions	1.67 (1.16-2.42)^b^	0.94 (0.73-1.20)	0.90 (0.79-1.03)	1.61 (1.14-2.26)^b^	3.43 (1.99-5.92)^b^
Presence of hypertension	0.93 (0.86-1.01)	0.93 (0.84-1.04)	0.94 (0.88-1.01	0.98 (0.91-1.04)	0.89 (0.79-1.01)

Abbreviations: ADG, Johns Hopkins Aggregated Diagnostic Groups; BMI, body mass index (calculated as weight in kilograms divided by height in meters squared); CABS, coronary artery bypass surgery; CAD, coronary artery disease; CKD, chronic kidney disease; ESKD, end-stage kidney disease; PCI, percutaneous coronary intervention; RYGB, Roux-en-Y gastric bypass.

^a^ The generalized estimating equations regression model for nonelective hospitalizations did not converge.

^b^ *P* < .05.

## Discussion

To our knowledge, this is the first study comparing long-term health care expenditures among patients undergoing RYGB vs sleeve gastrectomy. We found no statistically significant differences in 4-year overall health expenditures between matched cohorts of patients undergoing RYGB and sleeve gastrectomy. However, we found a positive association between sleeve gastrectomy and the 4-year costs associated with elective hospitalizations and a negative association with 4-year physician costs. We identified important patient-level factors associated with health care expenditures, such as history of chronic kidney disease, coronary artery disease, and mental illness admissions, which need further investigation to better understand the costs and outcomes associated with these groups of patients. Other results indicated that after 4 years, the 2 bariatric procedures did not differ significantly in terms of all-cause mortality and all types of rehospitalizations, but fewer subsequent bariatric procedures and more nonelective readmissions were associated with RYGB.

Our study is novel in several ways. First, compared with the observational literature derived from US studies,^6,7^ our study comparing RYGB and sleeve gastrectomy was conducted in Canada under a universal, publicly insured health care system. Second, we had access to several administrative databases, allowing us to examine health care expenditures beyond hospitalizations. This is important because our results showed that nonhospitalization costs accounted for approximately 50% of the total health care expenditures. We also observed differences in terms of elective vs nonelective readmissions. As such, studies focusing only on all-cause hospitalizations provide an incomplete view of health care expenditures after bariatric surgery. Third, as opposed to several recent studies using Cox proportional hazards regression models^6,7^ for the entire cohort of patients undergoing bariatric surgery, we used propensity scoring matching methods to create matched cohorts of patients receiving RYGB or sleeve gastrectomy.^22^ Although the external validity of the propensity score–matched results could be compromised if the matching process results in the exclusion of a large group of eligible patients, this was not the case in our study. We were able to match 85% of our sleeve gastrectomy cohort (812 of 926 eligible patients). Therefore, our results are generalizable to most sleeve gastrectomy procedures performed in Ontario.

Compared with a recent large observational study^7^ involving 33 560 patients from the US Patient-Centered Clinical Research Network who received RYGB (n = 18 056) or sleeve gastrectomy (n = 35 560) from January 1, 2005, to September 30, 2015, our Canadian cohort is slightly older (mean ages, 48.0 vs 45.0 years) and has a higher mean BMI (51.9 vs 49.1) but is otherwise similar. As in our study, this US study also reported low and similar mortality rates between RYGB and sleeve gastrectomy. Aligned with our study findings, the US data indicated that 2.8% of patients undergoing RYGB and 4.0% of patients undergoing sleeve gastrectomy underwent a revision by 5 years, when revision was defined as a conversion (eg, from sleeve gastrectomy to RYGB) or any revisional procedure (eg, gastrectomy).^7^ In contrast to the growing clinical literature comparing RYGB and sleeve gastrectomy, only a few comparative studies have evaluated health care expenditures associated with RYGB and sleeve gastrectomy, but their analyses were limited to 30 days after the index procedure.^23,24^

### Strengths and Limitations

Our study has several strengths. First, we had access to all publicly funded bariatric surgical procedures in Ontario and administrative databases accounting for all Ontario publicly funded health care expenditures. Therefore, we were able to compare RYGB and sleeve gastrectomy in terms of both inpatient and outpatient health care expenditures before and after the index procedures. We also used propensity score matching to compare sleeve gastrectomy and RYGB, thus providing a rigorous evaluation of the short- and long-term costs associated with RYGB and sleeve gastrectomy. Compared with registry-based studies with high attrition rates, we linked the Ontario Bariatric Registry data to administrative data bases, thus minimizing loss of follow-up. In our case, 97.4% of our cohort reached 48 months of follow-up.

Several limitations merit emphasis. First, we did not have access to medication costs, because prescription drugs for individuals younger than 65 years (ie, 1557 study patients [95.9%]) are generally not covered by public drug insurance. Second, although we were able to match study patients on many important baseline characteristics, there is always a risk for potential unmeasured confounding. However, we cannot think of confounders that are likely to be sufficiently prevalent to importantly alter our study findings. Third, we were unable to evaluate gastric banding because this procedure is not publicly reimbursed in Ontario. Fourth, owing to the small number of sleeve gastrectomy procedures conducted in Ontario until March 2015 (N = 926), we were unable to perform important subgroup analyses among groups such as patients with type 2 diabetes or a history of coronary artery disease. Although our mortality results are similar to those of other studies,^4,5,7^ our study was not powered to detect differences in mortality. Fifth, although we provided a detailed analysis of health care expenditures per cost components, it is difficult to directly compare the individual cost components because the cohorts were only matched in terms of overall health care expenditures in the 5 years before the index surgery. To provide further context, we used several multivariable regression models to compare expenditures between RYGB and sleeve gastrectomy in terms of hospitalizations, physician services, and other use of health care services. Although we separated elective vs nonelective hospitalizations in our analyses, we did not examine the underlying reasons for health care use and associated expenditures (eg, surgical complications vs elective joint replacement, which is now possible owing to weight loss after the bariatric procedure). This is an important avenue for future research. The results of this study are also based on an early cohort of sleeve gastrectomy performed from 2010 to 2015, and the generalizability of these results to more recent patients or those outside Ontario is unknown. Unfortunately, we did not have access to more recent data from the Ontario Bariatric Registry, which would have allowed us to document the outcomes associated with sleeve gastrectomy performed more recently (eg, during the last 5 years). Last, our comparison did not include other important patient-focused measures, such as indirect costs and health-related quality of life. This is important because although the procedures were associated with similar health care expenditures and mortality experience, they may have had different effects on quality of life, although evidence from randomized clinical trials suggests otherwise.^4,5^

## Conclusions

Our population-based analyses of patients from Ontario, Canada, indicate that 4-year health care expenditures, all-cause mortality, and subsequent hospitalizations did not significantly differ between cohorts undergoing RYGB and sleeve gastrectomy. Patients in the RYGB cohort underwent fewer subsequent bariatric procedures, whereas the number of subsequent nonelective admissions was higher with RYGB. Further investigation is needed to better understand the costs and outcomes after bariatric surgery among subgroups of patients who are at increased risk of health care expenditures.

## Notes

Supplement. **eTable 1.** Eligible Population **eTable 2.** Comparisons of Unmatched vs Matched Sleeve Gastrectomy **eTable 3.** Comparison of Unmatched vs Matched RYGB
