# Supplementary material for: Comparison of 4-Year Health Care Expenditures Associated With Roux-en-Y Gastric Bypass vs Sleeve Gastrectomy
Source: JAMA Netw Open. 2021 Sep 9;4(9):e2122079. doi: 10.1001/jamanetworkopen.2021.22079 (PMC8430456; doi:10.1001/jamanetworkopen.2021.22079)
Supplement: Supplement. — eTable 1. Eligible Population eTable 2. Comparisons of Unmatched vs Matched Sleeve Gastrectomy eTable 3. Comparison of Unmatched vs Matched RYGB [file jamanetwopen-e2122079-s001.pdf]

Supplementary Online Content

Tarride JE, Doumouras AG, Hong D, et al. Comparison of 4-year health care expenditures associated with Roux-en-Y gastric bypass vs sleeve gastrectomy. *JAMA Netw Open*. 2021;4(9):e2122079. doi:10.1001/jamanetworkopen.2021.22079

eTable 1. Eligible Population

eTable 2. Comparisons of Unmatched vs Matched Sleeve Gastrectomy

eTable 3. Comparison of Unmatched vs Matched RYGB

This supplementary material has been provided by the authors to give readers additional information about their work.

© 2021 Tarride JE et al. *JAMA Network Open*.

eTable 1. Eligible Population

	RYGB (N=6,301)	Sleeve (N=926)	Standardized Mean Difference
**Age**			
Mean (SD)	45.08 ± 10.27	48.36 ± 10.58	0.31
<45	3,047 (48.4%)	334 (36.1%)	0.25
45-54	1,993 (31.6%)	306 (33.0%)	0.03
55-64	1,149 (18.2%)	244 (26.3%)	0.20
65+	112 (1.8%)	42 (4.5%)	0.16
**Sex**			
Female	5,246 (83.3%)	692 (74.7%)	0.21
Male	1,055 (16.7%)	234 (25.3%)	0.21
**Body Mass Index**			
Mean (SD)	47.65 (6.93)	53.17 (9.67)	0.66
**Year of Index date**			
2010	93 (1.5%)	9 (1.0%)	0.05
2011	645 (10.2%)	101 (10.9%)	0.02
2012	1,658 (26.3%)	159 (17.2%)	0.22
2013	2,058 (32.7%)	313 (33.8%)	0.02
2014	1,529 (24.3%)	297 (32.1%)	0.17
2015	318 (5.0%)	47 (5.1%)	0.00
**Income Quintile**			
Lowest	1,407 (22.3%)	226 (24.4%)	0.05
2	1,455 (23.1%)	221 (23.9%)	0.02
3	1,309 (20.8%)	189 (20.4%)	0.01
4	1,228 (19.5%)	165 (17.8%)	0.04
Highest	885 (14.0%)	121 (13.1%)	0.03
Missing	17 (0.3%)	<=5	0.03

© 2021 Tarride JE et al. *JAMA Network Open*.

**Marginalization Index (summary score)**			
Mean (SD)	3.00 (0.75)	3.08 (0.73)	0.10
**Total number of major Johns Hopkins Aggregated Diagnostic Groups**			
Mean (SD)	5.96 (2.68)	6.93 (2.93)	0.35
**Select medical conditions in preceding 5 years**			
CRD / ESRD	113 (1.8%)	49 (5.3%)	0.19
CAD / PCI / CABS	826 (13.1%)	224 (24.2%)	0.29
Diabetes	2,129 (33.8%)	366 (39.5%)	0.12
Hypertension	2,765 (43.9%)	490 (52.9%)	0.18
Hypercholesterolemia	1,096 (17.4%)	146 (15.8%)	0.04
Mood and Anxiety Disorders Hospitalizations	92 (1.5%)	18 (1.9%)	0.04
**Healthcare expenditure in preceding 5 years (total)**			
Mean (SD)	$15,897 ($19,521)	$26,792 ($35,837)	0.38
**Healthcare utilization in preceding year**			
Mean (SD) number of days in hospital one-year prior index	0.24 (1.59)	0.64 (3.05)	0.17
Mean (SD) number of days in ED one-year prior index	0.68 (1.43)	0.92 (1.86)	0.15

Abbreviations: RYGB: Roux-en-Y-Bypass; SG: sleeve gastrectomy; SD: standard deviations; CRD: chronic renal disease; ESRD: end stage renal disease; CAD: coronary artery disease; PCI: percutaneous coronary intervention; CABS: coronary artery bypass surgery; N.A.: not applicable

© 2021 Tarride JE et al. *JAMA Network Open*.

eTable 2. Comparison of Unmatched vs Matched Sleeve Gastrectomy

	Unmatched Sleeve (N=114)	Matched Sleeve (N=812)	Standardized Mean Difference
**Age**			
Mean (SD)	50.41 (10.26)	48.07 (10.60)	0.22
<45	32 (28.1%)	302 (37.2%)	0.20
45-54	37 (32.5%)	269 (33.1%)	0.01
55-64	38 (33.3%)	206 (25.4%)	0.18
65+	7 (6.1%)	35 (4.3%)	0.08
**Sex**			
Female	71 (62.3%)	621 (76.5%)	0.31
Male	43 (37.7%)	191 (23.5%)	0.31
**Body Mass Index**			
Mean (SD)	62.36 (9.77)	51.88 (8.93)	1.12
**Year of Index date**			
2010	0 (0.0%)	9 (1.1%)	0.15
2011	13 (11.4%)	88 (10.8%)	0.02
2012	11 (9.6%)	148 (18.2%)	0.25
2013	37 (32.5%)	276 (34.0%)	0.03
2014	47 (41.2%)	250 (30.8%)	0.22
2015	6 (5.3%)	41 (5.0%)	0.01
**Income Quintile**			
Lowest	32 (28.1%)	194 (23.9%)	0.10
2	30 (26.3%)	191 (23.5%)	0.06
3	20 (17.5%)	169 (20.8%)	0.08
4	16 (14.0%)	149 (18.3%)	0.12
Highest	12 (10.5%)	109 (13.4%)	0.09

© 2021 Tarride JE et al. *JAMA Network Open*.

Missing	<=5	0 (0.0%)	0.27
**Marginalization Index (summary score)**			
Mean (SD)	3.15 (0.65)	3.07 (0.74)	0.12
**Total number of major Johns Hopkins Aggregated Diagnostic Groups**			
Mean (SD)	7.87 (3.20)	6.80 (2.87)	0.35
**Select medical conditions in preceding 5 years**			
CRD / ESRD	19 (16.7%)	30 (3.7%)	0.44
CAD / PCI / CABS	58 (50.9%)	166 (20.4%)	0.67
Diabetes	56 (49.1%)	310 (38.2%)	0.22
Hypertension	77 (67.5%)	413 (50.9%)	0.34
Hypercholesterolemia	13 (11.4%)	133 (16.4%)	0.14
Mood and Anxiety Disorders Hospitalizations	<=5	15 (1.8%)	0.05
**Healthcare expenditure in preceding 5 years (total)**			
Mean (SD)	$50,760 ($63,741)	$23,428 ($28,401)	0.55
**Healthcare utilization in preceding year**			
Mean (SD) number of days in hospital one-year prior index	1.69 (5.06)	0.50 (2.62)	0.30
Mean (SD) number of days in ED one-year prior index	1.04 (1.82)	0.91 (1.86)	0.07

Abbreviations: RYGB: Roux-en-Y-Bypass; SG: sleeve gastrectomy; SD: standard deviations; CRD: chronic renal disease; ESRD: end stage renal disease; CAD: coronary artery disease; PCI: percutaneous coronary intervention; CABS: coronary artery bypass surgery

© 2021 Tarride JE et al. *JAMA Network Open*.

eTable 3. Comparison of Unmatched vs Matched RYGB

	Unmatched RYGB (N=5,489)	Matched RYGB (N=812)	Standardized Mean Difference
**Age**			
Mean (SD)	44.66 (10.17)	47.90 (10.56)	0.31
<45	2,741 (49.9%)	306 (37.7%)	0.25
45-54	1,726 (31.4%)	267 (32.9%)	0.03
55-64	942 (17.2%)	207 (25.5%)	0.20
65+	80 (1.5%)	32 (3.9%)	0.15
**Sex**			
Female	4,625 (84.3%)	621 (76.5%)	0.20
Male	864 (15.7%)	191 (23.5%)	0.20
**Body Mass Index**			
Mean (SD)	47.02 (6.48)	51.91 (8.28)	0.66
**Year of Index date**			
2010	85 (1.5%)	8 (1.0%)	0.05
2011	549 (10.0%)	96 (11.8%)	0.06
2012	1,526 (27.8%)	132 (16.3%)	0.28
2013	1,773 (32.3%)	285 (35.1%)	0.06
2014	1,279 (23.3%)	250 (30.8%)	0.17
2015	277 (5.0%)	41 (5.0%)	0
**Income Quintile**			
Lowest	1,240 (22.6%)	167 (20.6%)	0.05
2	1,244 (22.7%)	211 (26.0%)	0.08
3	1,136 (20.7%)	173 (21.3%)	0.01
4	1,082 (19.7%)	146 (18.0%)	0.04
Highest	770 (14.0%)	115 (14.2%)	0.00
Missing	17 (0.3%)	0 (0.0%)	0.08

© 2021 Tarride JE et al. *JAMA Network Open*.

**Marginalization Index (summary score)**			
Mean (SD)	3.00 (0.75)	3.04 (0.74)	0.06
**Total number of major Johns Hopkins Aggregated Diagnostic Groups**			
Mean (SD)	5.83 (2.62)	6.81 (2.88)	0.35
**Select medical conditions in preceding 5 years**			
CRD / ESRD	87 (1.6%)	26 (3.2%)	0.11
CAD / PCI / CABS	670 (12.2%)	156 (19.2%)	0.19
Diabetes	1,819 (33.1%)	310 (38.2%)	0.11
Hypertension	2,353 (42.9%)	412 (50.7%)	0.16
Hypercholesterolemia	961 (17.5%)	135 (16.6%)	0.02
Mood and Anxiety Disorders Hospitalizations	85 (1.5%)	7 (0.9%)	0.06
**Healthcare expenditure in preceding 5 years (total)**			
Mean (SD)	$ 15,109 (17,835)	$21,220 (27,841)	0.26
**Healthcare utilization in preceding year**			
Mean (SD) number of days in hospital one-year prior index	0.21 (1.48)	0.48 (2.17)	0.15
Mean (SD) number of days in ED one-year prior index	0.64 (1.32)	0.91 (1.99)	0.16

Abbreviations: RYGB: Roux-en-Y-Bypass; SG: sleeve gastrectomy; SD: standard deviations; CRD: chronic renal disease; ESRD: end stage renal disease; CAD: coronary artery disease; PCI: percutaneous coronary intervention; CABS: coronary artery bypass surgery; N.A.: not applicable

© 2021 Tarride JE et al. *JAMA Network Open*.
